# Individually Unique Body Color Patterns in Octopus (*Wunderpus photogenicus*) Allow for Photoidentification

**DOI:** 10.1371/journal.pone.0003732

**Published:** 2008-11-14

**Authors:** Christine L. Huffard, Roy L. Caldwell, Ned DeLoach, David Wayne Gentry, Paul Humann, Bill MacDonald, Bruce Moore, Richard Ross, Takako Uno, Stephen Wong

**Affiliations:** 1 Monterey Bay Aquarium Research Institute, Moss Landing, California, United States of America; 2 Department of Integrative Biology, University of California, Berkeley, California, United States of America; 3 New World Publications, Jacksonville, Florida, United States of America; 4 Dallas, Texas, United States of America; 5 Bill Macdonald Productions, Venice, California, United States of America; 6 Black Sand Dive Retreat, Kasawari, Lembeh Strait, North Sulawesi, Indonesia; 7 Steinhart Aquarium, San Francisco, California, United States of America; 8 Homantin, Kowloon, Hong Kong; University of Exeter, United Kingdom

## Abstract

Studies on the longevity and migration patterns of wild animals rely heavily on the ability to track individual adults. Non-extractive sampling methods are particularly important when monitoring animals that are commercially important to ecotourism, and/or are rare. The use of unique body patterns to recognize and track individual vertebrates is well-established, but not common in ecological studies of invertebrates. Here we provide a method for identifying individual *Wunderpus photogenicus* using unique body color patterns. This charismatic tropical octopus is commercially important to the underwater photography, dive tourism, and home aquarium trades, but is yet to be monitored in the wild. Among the adults examined closely, the configurations of fixed white markings on the dorsal mantle were found to be unique. In two animals kept in aquaria, these fixed markings were found not to change over time. We believe another individual was photographed twice in the wild, two months apart. When presented with multiple images of *W. photogenicus*, volunteer observers reliably matched photographs of the same individuals. Given the popularity of *W. photogenicus* among underwater photographers, and the ease with which volunteers can correctly identify individuals, photo-identification appears to be a practical means to monitor individuals in the wild.

## Introduction

Despite the large number of octopuses recognized from the Indo-Pacific [1] and the importance of cephalopods to tropical food webs and fisheries [2] very little is known about their home ranges, population densities, and natural survivorship. Among the many challenges to obtaining such data is the difficulty of tracking individuals over time in the wild. In the past, researchers have used naturally occurring injuries to identify individuals [3], [4]; however this method does not permit long-term identification because arms regenerate and most injuries heal. Octopuses are flexible enough to pull out many types of external tag, rendering useless many of the means used to track other cephalopods such as squids [5]. External tags can fall out on their own [6], and those that involve electronics are limited by cost, geographic range and battery power [7], [8]. These techniques, along with artificial markings such as branding and tattooing, are also invasive and/or require the animal to be handled. While informative for robust octopuses, most tracking methods are not practical for use with small or delicate species [5]. Methods for using growth rings of stylets (hard structures in the mantle muscle) to assess longevity of wild octopuses are improving, but are rarely validated [but see 9] and require that the animal be sacrificed.

For many animal groups, variation in naturally occurring body color markings is used to identify individuals. This tool is an inexpensive and non-invasive means to study survivorship, intra-specific behavioral interactions, population estimates, and large-scale migration patterns of wild animals, as in cetaceans [10]; coelacanths [11]; cheetahs [12]; and whale sharks [13]. Body color patterns are particularly important when studying animals that lack hard structures, such as antlers, that help researchers identify some vertebrates [14]. Photoidentification allows populations of animals to be sampled without handling or extracting individuals, which is necessary if they are delicate, rare and/or commercially important to ecotourism. For this method to be successful, body color pattern must vary across individuals, but remain unchanged for any individual over time. This concept is well established among biologists and conservationists studying vertebrates, but is seldom used in studies of invertebrates. Good examples of how unique body color patterns can be used to follow individual invertebrates are studies on individual recognition in arthropods [15], [16]. To our knowledge body color patterns have not yet been used to identify individual octopuses, perhaps because the skin appearance in many species changes so rapidly.

As in other animals, body patterns in octopuses are constrained by a fixed skin anatomy [17], [18]. Although their intensity and texture can vary considerably based on individual expression, the location of skin components and the range of pigments appear to be species-specific [19]. For example the presence or absence of false eyespots, ‘dorsal mantle white spots’, eye ornamentation, and ‘lateral neck dark spots’ are among many skin characteristics that greatly facilitate taxonomic identification in this group, particularly when examining photographs of live animals [19], [20].

**Figure 1:**
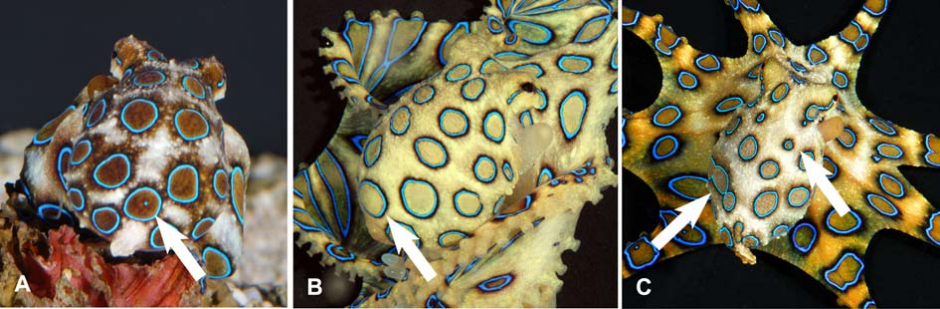
Variable ring patterns on mantles of the blue-ringed octopus *Hapalochlaena lunulata*. Note the small fleck of blue in the ring indicated in panel A, which is missing from the corresponding ring in panel B. The individual in panel C bears disproportionally small rings near the head, as well as merged rings left side. All photographs by Roy Caldwell.

While intra-specific differences in skin anatomy may be difficult to identify in species with complex skin, they can exist. With among the richest body pattern repertoires of any octopus, individual *Abdopus aculeatus* have been noted to vary consistently in their expression of body patterns during defense [21]. The blue-ringed octopus *Hapalochlaena lunulata* exhibits variable patterns of its highly-visible rings (Figure 1). The delicate octopus *Wunderpus photogenicus* exhibits a relatively limited body pattern repertoire consisting of fixed white markings on a rusty brown background [22]. While examining photographs of this octopus we noticed that the shape and position of these markings differ among individuals possibly providing a means to identify individuals and track them over time and distance.

*Wunderpus photogenicus* is a charismatic tropical octopus that is commercially important to the underwater photography, dive tourism, and home aquarium trades. However, individuals and populations have yet to be monitored in the wild. In the Lembeh Strait, Indonesia, a well-established tourist destination for tropical soft-sediment (“muck”) diving, *W. photogenicus* is now among the two animals most sought-after by underwater photographers (B. M., personal observation). As the commercial importance of this species to dive tourism grows, so does demand for the aquarium trade (www.Tonmo.com). This octopus is an expensive marine ornamental, with suppliers citing rarity and beauty as the reasons for prices upwards of $700. Populations appear to be highly variable, fluctuating between extreme rarity (none seen for four months, B.M. personal observation) and densities of up to 5 individuals per 25 m^2^ (C.L.H., personal observation). Variability in abundance, longevity, and movement patterns remain unstudied. With no monitoring information about *W. photogenicus* currently available to management agencies, and given the value of live animals to ecotourism, a non-extractive means to identify and track individual adults is currently needed.

Studies of *Wunderpus photogenicus* could benefit greatly from photoidentification because their small size and delicate body rule out using currently available tagging methods to monitor stocks. Here we describe the mantle white marking patterns of multiple adult *Wunderpus photogenicus*, offering a means to identify individual adults. Underlying the utility of photoidentification as a monitoring tool is the ability of either human observers [10] or computer programs [13] to pair an organism correctly with photographs taken of that individual. Thus we also demonstrate the ability of volunteers to correctly match multiple views of an individual when presented with a series of *W. photogenicus* images.

## Methods

We solicited photographs from fifteen underwater photographers known to document *W. photogenicus* in the wild, as well as people known to have kept them in aquaria. We had specifically requested images taken of the dorsal mantle from above because this view allows easy comparison of spot patterns, and it can be photographed easily.

**Figure 2:**
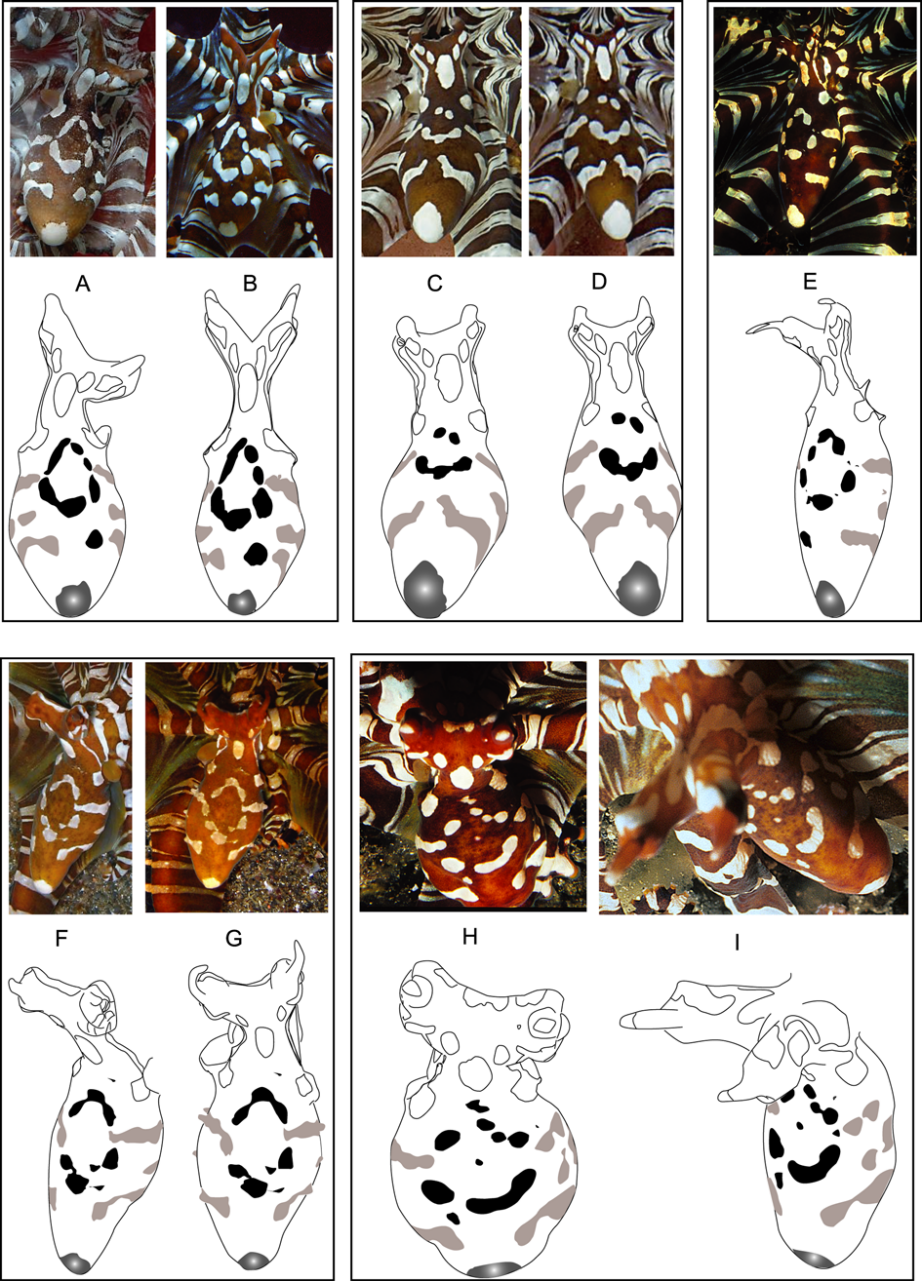
Configuration of white markings on the dorsal mantle of five individual *Wunderpus photogenicus*. Outlines indicate which photographs were taken of the same individual. Underneath each photograph is the corresponding outline of mantle markings: central white spots in black, side markings in grey, posterior mantle spot grey with faded center. [photographs by: A–D. Richard Ross (animals A–B and C–D each from the home aquarium trade); E. Takako Uno (North Sulawesi, Indonesia); F.–G. CLH (North Sulawesi, Indonesia); H–I. Roy Caldwell (North Sulawesi, Indonesia).]

Images depicting the dorsal mantle of *W. photogenicus* from directly above proved to be rare in photographic collections. From our search we obtained 30 high quality photographs and video frames taken of *Wunderpus photogenicus* in their natural habitat (n = 13 individuals from Indonesia and the Philippines), and in a home aquarium (n = 2 individuals). Individuals in which founder chromatophores were visible were considered juveniles and were not examined. Founder chromatophores are pigment sacs in the skin that tend to be more prominent in juvenile octopuses, but become obscured in adults as the skin completes its development [18]. Six of these adults were photographed from directly above to provide clear views of the dorsal mantle. Five of these individuals are depicted in Figure 2, and the sixth has been published previously [22]. Multiple images were available for four of these individuals. These images were taken at intervals ranging from approximately ten minutes (Figure 2F–G, H–I) to 10.5 weeks (Figure 2A–B). Photographs taken from a slightly oblique angle allowed sufficient comparison for the purposes of survey Group A described below.

White markings on the dorsal mantle were traced in Adobe Illustrator CS2, and a diagram produced in order to visualize variations between these markings among individuals. The markings of interest were depicted in black in these diagrams (Figure 2). Although white markings were visible during crypsis, their outlines were not consistently distinct. Additionally, erection of the papillae appeared to modify the shape of white markings slightly. Thus images of camouflaged individuals and individuals with strongly erect papillae were not analyzed.

### Photoidentification

We tested the accuracy with which observers matched multiple photographs of individual *Wunderpus photogenicus*. Volunteer observers were asked to examine the white markings on the center of the mantle to determine whether they felt body patterns on multiple panels matched each other. Observers were not told if or how many matches were included. Group A (n = 11) was presented with a 24 panel collage of photographs which included one to four panels of each *W. photogenicus*. Observers in this group then provided us a written list of panels that “matched”. Groups B and C (each n = 11) were presented with 50 PowerPoint slides, each depicting two images. In addition to these images, slides presented to Group C also included an outline of body patterns for each depicted *W. photogenicus* image, as exemplified in Figure 2. Observers in Groups B and C indicated electronically whether these images were a “match” (both panels depicted the same individual octopus) or “no match” (each panel depicted different individual octopuses). “Match” and “No Match” were indicated in text using blue and orange text boxes respectively to allow ease in scoring using the “Slide Sorter” option of PowerPoint. Each observer was given a score based on how many *W. photogenicus* photographs were correctly identified as matches or mis-matches, and results are presented as percentages of possible points. For groups B and C we also compared erroneous matches (observers incorrectly identified images of two different individual *W. photogenicus* as matching) and missed matches (observers incorrectly identified images of the same individual *W. photogenicus* as not matching). Statistical analyses of resulting data were conducted using StatXact 4.0.1.

## Results

Each adult *Wunderpus photogenicus* examined (n = 15) exhibited a distinct configuration of white markings on the dorsal mantle (examples illustrated in Figure 2). Most of this variation took place among the white markings on the central dorsal mantle rather than the consistent markings on the head, neck, and posterior tip of the mantle. Each animal bore a circular pattern of approximately six white spots in the center of the mantle. However fusions of these spots and the location of additional small markings in this region differed among individuals. Lateral markings also appeared to vary asymmetrically. While using them alone to identify individuals was problematic because standardized views of left and right sides were not available for direct comparison, they provided valuable supplemental information to the central mantle markings.

### Photoidentification

When matching multiple images of *W. photogenicus* 59% of all participants scored above 90%, with 36% scoring higher than 95%. Overall, the three test groups yielded similar test results (Total score 82±3%S.E.; ANOVA F = 1.2, p = 0.5). However participants in Group A indicated having difficulties with the testing format.

**Figure 3:**
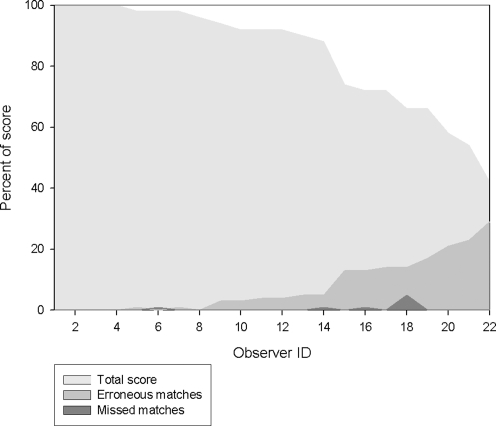
Scores of each observer in Groups B and C, illustrating erroneous and missed matches when assessing images individual *Wunderpus photogenicus*.

Because they yielded similar scores and because their formats were considered similar (50 slides, each comparing two images), groups B and C were combined to examine whether participants were more likely to give erroneous or missed matches. This analysis did not include Group A because that test format was considerably different (comparing multiple image panels on a single page), had a very high number of potential erroneous matches, and because we do not plan to use it in the future. In groups B and C, missed matches were rare (Figure 3); on average participants missed 4.5±2% S.E. of total possible matches. Erroneous matches were more common (18±4% S.E. of non-matching individuals were erroneously assessed as a match), and strongly correlated with overall score (Spearman's CC = −0.9759, p<0.0001).

## Discussion

Each of the adult *Wunderpus photogenicus* examined demonstrated a unique arrangement of the fixed white markings on the dorsal mantle. We do not believe that this variation reflects sexual dimorphism, ontogenetic shifts, or artifacts of mantle distortion. We observed a unique pattern for each individual, rather than only two patterns total as would have been expected if this species exhibited male-typical and female-typical spot configurations. Additionally, while body patterns are known to become more complex throughout the life of an octopus [23] to our knowledge fixed skin components are not known to change location or expand significantly into one another in adulthood, as would be necessary to impact our results. Volunteers consistently matched multiple images of an individual *W. photogenicus* taken up to 10.5 weeks apart. Finally, several lines of evidence suggest that this variation is not an observational artifact resulting from varying degrees of mantle distortion. 1) All photos except one appear to have been taken when the mantles were similarly relaxed, as between ventilations; 2) the single expanded mantle image was consistently paired with its relaxed counterpart (Figure 2H–I) by volunteers; 3) mantle expansion and contraction with ventilation should cause uniform magnification and reduction of the entire pattern rather than other types of distortion; 4) we analyzed photographs with minimal papillae erection to minimize artifacts of white spot distortion with changes in skin texture. Thus although the distance between distinct spots may vary slightly with mantle expansion, white spots would not be expected to separate or fuse, or change shape significantly during breathing.

Untrained observers were able to differentiate individual *W. photogenicus* based on photographed body patterns. Although observers from all groups performed equally well overall, some individuals were considerably better than others at finding correct matches. Given the accuracy with which these volunteers matched individual *W. photogenicus*, researchers should have no problem pre-screening participants and finding skilled people to help monitor collections of images. By doing so, researchers should be able to minimize erroneous matches, which would lead us to underestimate population size and overestimate longevity and dispersal. Additionally, because the populations are believed to be relatively small compared to other animals that are monitored by using photoidentification, we do not expect that there will be an unmanageable number of photographs to sort.

*Wunderpus photogenicus* is an ideal candidate for the use of photoidentification in ecological studies of octopuses. Because these animals are already sought after by underwater photographers, efforts to initiate this monitoring program are likely to be met with enthusiasm among the diving community. By collaborating with underwater photographers, scientists may be able to use individual spot patterns to track individuals at popular dive sites and monitor both longevity and small scale movement patterns. If the variation in the color patterns observed here indeed represents unique marking patterns of adults across populations of *W. photogenicus*, then we believe it will be possible to recognize individuals over geographic and time scales greater than is feasible with methods currently used to track wild octopuses. For example, our collection includes photographs taken in Milne Bay (Papua New Guinea) in November 1991 and January 1992. Based in similarities in the spot patterns, we believe these photographs represent the same individual *W. photogenicus* documented in the wild at a two-month interval. By creating a database of photographs we even may be able to identify individuals harvested from protected areas and subsequently sold in the aquarium trade. To initiate this effort we urge underwater photographers to consider taking photographs of *W. photogenicus* mantles from directly above and deposit them in the “Wunderpix” database of the website 〈http://calphotos.berkeley.edu/Wunderpix.html〉. This information will be distributed to dive operators throughout the range of *W. photogenicus* as well as posted on internet venues frequented by underwater photographers, home aquarists, and cephalopod enthusiasts.

The list of animals with individually recognizable (by humans) markings is growing. However, our discovery of such markings in an octopus came as a surprise. These animals are well-known for their ability to vary their color pattern and skin texture, producing patterns so complex that individual markings have hitherto been considered difficult or impossible to distinguish. Additionally, in at least *Polistes* wasps, markers of identity are disproportionally prominent in social taxa, suggesting that in some cases they may be naturally selected and maintained in lineages with repeated interaction and the need to differentiate conspecifics [24]. Individual recognition has not been demonstrated in any octopus, as this concept has not been tested rigorously [25]. These animals have traditionally been considered asocial, and so are unlikely to have evolved means for individual recognition [25]. However we cannot discount the possibility that it occurs in these animals with high visual acuity and the ability to remember [26].
